# Ergot

**Published:** 1875-11

**Authors:** T. Curtis Smith

**Affiliations:** Middleport, Ohio


### ERGOT.

BY T. CURTIS SMITH, M. D., MIDDLEPORT, OHIO.

This agent has, within the past half decade, been so much written up by medical authorities, high and low, and in all countries where scientific medicine is known, that it might seem a work of supererrogation to attempt anything further on the subject. But I am led to believe that its use in some directions is not founded in good reason, and that it is lauded too highly for some difficulties, while, on the other hand, it is not impossible that a further knowledge of its valuable powers may be wanting in some directions. In this paper, I shall not attempt to go over all the ground of the subject, and shall not more than hint at its uses and powers during gestation and parturition, as these points are so fully set forth in all standard works on these topics.

It is but natural to first inquire what are the physiological effects of ergot, in order that we may deduce from a knowledge of such effects what its therapeutic application would be when its use is demanded at the bedside.

Ergot, it seems, possesses at least two active principles, which we shall notice without special reference to other principles it is supposed to contain. These are the alcoholic and watery extracts of ergot. One of these is soluble in alcohol, but not in water, while the reverse is the case with the other. Wiggers was probably the first to procure the alcoholic extract, while Bonjean first brought forward the watery extract, to which only the term ergotine is properly applied. The chemical fact that these extracts were not each soluble in alcohol or water led to the supposition that the physiological effects of the two extracts might be quite different, and such has been proven to be the case. This has been a very fruitful source of discrepancy in the effects of the so-called ergotine, which, in very many experiments on animals and man, has been entirely overlooked. This fact being now prominently brought forward and demonstrated by such able authorities as Anstie, Handelin, Kohler, Hilderbrandt, and others, it is to be hoped that more uniform results will follow in practice than has heretofore been the case.

Bonjean's ergotine—the watery extract—stimulates the depressor centers of the heart, and vasomotor centers of the medulla oblongata, thereby producing slowness of pulse and contraction of the arteries with consequent increase of blood-pressure. [Kohler.] Large doses paralyze the heart. The alcoholic extract has no influence over the heart or blood-vessels, but will, in large doses, produce irritation of the mucous membranes, and serious poisonous effects. The aqueous extract reduces peripheral and motor irritability, when acting directly upon them, while the alcoholic increases it. Both extracts reduce the respiration and temperature, but the alcoholic more than the aqueous. Both cause dilatation of the pupil and diminish the irritability of the sensory nerves. From these facts we must conclude that a discrimination must be made in the therapeutic application of these agents or extracts. When we desire to secure contraction of the blood-vessels, to slow the heart's action, reduce temperature, and reflex action, then the aqueous extract should be administered. The alcoholic extract is useless as an hæmostatic, and, unlike the aqueous extract, it increases instead of depresses the irritability of peripheral motor nerves. These are points worthy of remembrance in prescribing an extract of ergot, and, as before stated, will account in some measure for the varying results obtained from ergotine as we find it in the market, and from fluid extracts. I have often heard practitioners say that they had given fluid extract ergot in half-ounce doses in cases of tedious labor, without causing any perceptible increase in the force of the uterine contractions. I have likewise known large quantities of the infusion given without apparent effect, but not always so. But I do not remember an instance where a drachm, or two drachms, of the powdered ergot, of good quality, was given in cases of labor, that the uterus did not respond to its action. May or may not these varying effects be due to the facts set forth in regard to the action of the different extracts, for in the fluid extract and the infusion we get but the portion of active principle that will be dissolved in water, while the other active portion can only be extracted by alcohol.

I have brought these points out this early in the paper in order that we may keep them before our minds in the study of its action, and also for the reason that we now, for the most part, prescribe the extracts, in one or other form, rather than the powdered ergot.

Small doses of ergot, given in health to the male or non-pregnant woman, produce no perceptible effects on the economy. I will give it as my opinion, based upon personal observation, that it will not, even in large doses, in substance or extract, produce any effect over the uterus calculated to lead to abortion, in the pregnant woman, during the first five or six months of gestation; and, in one case, where for other reasons I gave it largely during the eighth month, no uterine contractions followed, and the lady went on to full term and was delivered of a healthy male child. I am therefore satisfied that it does not produce the bearing-down pains in non-pregnant women and in early pregnancy, that many of our older authorities speak of. In short, it will not originate uterine contractions *de novo.* Ergot, given in ʒi to ii repeated doses, either to male or female, will be likely to cause more or less nausea, some cerebral disturbance, with dilatation of the pupil, slight narcosis, and diminished force and frequency of the heart's action ; the caliber of the blood-vessels, at least the smaller ones, become diminished by its action on the unstripped muscular fiber. (Brown Lequard.) If the dose be very large, the cerebral disturbance becomes more manifest in slight giddiness, heaviness, and a feeling constriction about the head, sometimes amounting to a most acute pain. If given when the brain is not sufficiently supplied with blood, these symptoms soon become manifest and painful. Pressed still farther, irregular and involuntary contractions occur, but this effect is not uniformly noticeable. There is not usually any disturbance of vision, except that a strong light becomes at once painful and difficult to endure.

Handelin states that the aqueous extract will, according to the dose, used hypodermically, cause anæsthesia, incoördination, paralysis, both voluntary and reflex, and that these are due to its effects over the nerve-centers—not over the peripheral nerves.

There is nearly uniform testimony as to the power of ergot, and its aqueous extracts, to produce diminished heart-action, diminished caliber of the blood-vessels, and depression of the nervous system, through its power over the nerve-masses, while it leaves the peripheral nerves without very well marked effects.

Of its poisonous effects, when long continued, much has been said, and the fact is notorious that in countries where the inhabitants are large consumers of rye, that dry gangrene not infrequently results from its use. Ergot has been continued for months in the treatment of disease without any such effects being observed. I gave it to one of my patients for four months, continuously, without noticing any other than apparently beneficial effects from its use, and have often continued it for one and two months continuously, no ill effects following.

The lethal dose of ergot is probably not known, as no cases of death from its administration are recorded, as far as I know.

Like many other medicines in common use, its power over the system is more effectually obtained by its hypodermic use.

Therapeutic application, from the now known physiological effects of ergot, we must naturally infer that it has a wide range of application in the treatment of diseases. Yet, I doubt very much whether ten years hence it will be applied in the treatment of as many and varied forms of disease as it is now commended for. In other words, in a few years it will find its level as a most valuable remedy, which it will ever maintain and not again be subject to fluctuations, such as those through which it has been and is now passing.

Prominent among its valued powers is the well established influence it has over the circulation of the brain and spinal cord and their meninges. Inflammation and congestion of these structures must ever stand among the grave diseases, and have allotted to them a large fatal list. Yet, I am sanguine, that as the agent in question becomes more thoroughly known in its power over these diseases, the mortality list will fall considerably short of what it has been in the past.

In cerebro-spinal meningitis, spinal meningitis and inflammation of the cerebral meninges, also in congestion of these membranes, it is fast becoming a reliable and standard remedy. It is also assuming the same ground in relation to structural inflammation of the brain, medulla and cord, and the degree of success reported with its use, in these diseases, exceeds by far that which has followed any other plan of treatment. In the few cases coming under my observation, it has afforded better results than I felt I had any reason to expect. In the treatment of cephalalgia caused by, or accompanied with, a determination of blood to the head, it is a most valuable agent, and often for me afforded relief where anodynes, hypotics, alteratives and other agents had failed. But if administered in a case where cerebral anæmia already existed, the pain and cerebral disturbance has quite generally been greatly increased. For this reason the cases for its use require selection. In mental diseases, J. Crichton Browne, Medical Director West Riding Asylum, states that for six years (*American Journal of Medical*

*Science*, October, 1871,) he has made an extensive series of experiments with ergot of rye in the treatment of various forms of insanity, and has arrived at results which he believes to be of considerable practical importance. He has found it valuable in recurrent mania; in chronic mania with lucid intervals, and in epileptic mania. He says: "In these forms of cerebral derangement, I have found it almost uniformly efficacious in reducing excitement, in shortening attacks, in widening the intervals between them, occasionally in altogether preventing their recurrence, and averting that perilous exhaustion by which excitement is so often succeeded." The influence it has in these diseases, Dr. Browne believes to be due to its power to diminish the size of the cerebral blood-vessels, thus cutting off the excessive supply of blood to the brain in a great measure.

Its power over the dimensions of the blood-vessels has led to its use as an hæmostatic. It has now been used successfully in pulmonary hemorrhages, epistaxis hæmatemesis, hematuria, intestinal hemorrhage, and is even reported as successful in checking persistent hemorrhage from a deep incised wound. For all these difficulties, except the last, I have used the agent with good success, except in a single instance in a case of bleeding from the lungs, which persisted in spite of this agent, but which yielded to large doses of quinine. In hemorrhage from ulcer of the stomach, and the ulcerations that accompany typhoid fever, as well as intestinal bleeding from the pupuric state of the blood, it has acted admirably for me in every instance. That it will invariably do this well, my experience has not been sufficient to warrant me in saying. But there is no lack of authorities in this and other countries to establish the hæmostatic properties of ergot in all passive hemorrhages. Its value in the passive forms of uterine hemorrhage has too long been well known to need more than mention here, and as an adjuvant in active uterine hemorrhage its value is well known.

In hæmoptisis of phthisis, Anstie and Currie Ritchey, of England, have spoken strongly in its favor as compared with plumbi acet., gallic acid, alum per sulph. of iron, etc. In their hands, while it did not control the flow of vital fluid in every instance, it nevertheless gave a good proportion of results. This is as much as can be said of any agent in general use for this difficulty.

In hemorrhoids the full value of the agent is not yet known, but it is now becoming well enough commended to secure general trial in this direction, and is already highly extolled by a few eminent medical men. In my own practice, I have resorted to its use in but two cases. These were both extreme cases of the trouble, as in both instances the hemorrhoids were large, and numbered ten in one and thirteen in the other. These came down at every passage from the bowels and would remain outside the anus until pushed back, sometimes becoming so completely strangulated as to remain down for days, causing great pain, annoyance and inconvenience to the patients. As in neither case could I secure consent to their removal, I resorted to injections twice daily of dram doses of fluid extract of ergot, without much confidence of success. But in a few days after commencing this treatment, the piles became much smaller, less painful and ceased to show themselves externally when the bowels were moved. I thought both cases might have been permanently cured by this method, but, like many of our cases, when they are relieved sufficiently and get so they suffer but little annoyance, they consider the further use of medicines a great bore, and hence stop them. Neither of these cases now suffer any inconvenience from the disease, though it has been six and nine months respectively since they were treated.

In prolapsus ani this agent is highly commended by Von Leangenbeck, of Germany, and others of England and this country report remarkable success.

In vavix and varicocele, it is highly commended by a few, but the success in these directions has not been as good as at first expected.

In the treatment of the pulmonary and throat diseases, such as chronic pharyngitis and laryngitis, chronic bronchitis and longstanding hepatization from pneumonia, it is well spoken of by a few German authorities. In these difficulties I found it, in some cases, to far surpass my own expectations, and cases under its use have completely recovered, that had long resisted other and well approved plans of treatment. I have no reason to doubt that it would prove highly serviceable in cases of enlarged bronchial glands, with which we occasionally meet. Whether it would be of any value in phthisis in any stage, I am unable to state, but would not expect benefit.

It is well commended in enlargements of the spleen and diabetes insipidis, but these instances require further confirmation.

In uterine fibroids many authorities now commend it in high terms, while a few speak doubtfully of its power.

When the rage in the use of ergot has once fairly subsided, it will probably have few high laudations, but will stand upon a solid basis more perfectly understood than now, probably be used in a more limited field, but used with a certainty of expected results, equal to our expectancy of results from quinia, morphia, etc. Meantime, let us watch and wait for such time to come in its history and use.

It will be noticed that in the entire list of diseases for which this agent has recently been brought forward, the same ends are indicated more or less prominently, namely, diminution in the caliber of the blood-vessels, diminished frequency and force of heart-action, and depression of the nervous system. Just what effect the agent has over the glands of the system, I am not now prepared to state, but such action is strongly claimed, and may be easily inferred from the effects claimed for it in some diseased conditions treated with it by leading men in this and other countries.

